# CD24 for Cardiovascular Researchers: A Key Molecule in Cardiac Immunology, Marker of Stem Cells and Target for Drug Development

**DOI:** 10.3390/jpm11040260

**Published:** 2021-04-01

**Authors:** Eyal Sagiv, Michael A. Portman

**Affiliations:** Division of Pediatric Cardiology, Seattle Children’s Hospital and the University of Washington School of Medicine, Seattle, WA 98105, USA; michael.portman@seattlechildrens.org

**Keywords:** CD24, dilated cardiomyopathy, vasculitis, immune modulator, multisystem inflammatory syndrome in children (MIS-C)

## Abstract

The study of the membrane protein, CD24, and its emerging role in major disease processes, has made a huge leap forward in the past two decades. It appears to have various key roles in oncogenesis, tumor progression and metastasis, stem cell maintenance and immune modulation. First described in the 1980s as the homologous human protein to the mouse HSA (Heat Stable Antigen), it was reported as a surface marker in developing hematopoietic cell lines. The later discovery of its overexpression in a large number of human neoplasms, lead cancer researchers to discover its various active roles in critical checkpoints during cancer development and progression. Targeting CD24 in directed drug development showed promising results in cancer treatment. More recently, the chimeric CD24-Fc protein has shown exciting results in clinical trials as a specific modulator of auto-inflammatory syndromes. This report is aimed to summarize the relevant literature on CD24 and tie it together with recent advancements in cardiovascular research. We hypothesize that CD24 is a promising focus of research in the understanding of cardiovascular disease processes and the development of novel biological therapies.

## 1. Background

CD24 is a membrane protein that is composed of a short peptide backbone with no transmembrane domain and is anchored to the membrane by glycosyl-phosphatidyl-inositol (GPI) [1]. It is heavily glycosylated in a pattern that can change between different cells, perhaps accounting for its various interactions and roles. The human *CD24* gene is located on chromosome 6q21. The initial cloning and mapping of the *CD24* gene was challenging due to the presence of homologous areas in the human genome, especially two highly observed sequences in chromosome location 15q21 and Yq11, most likely non-coding *pseudo*-genes [2]. The conservation of gene copies in human genome, highly homologous to its sequences in other species, is suggestive of a functional role with evolutionary significance. The human gene encodes a long mRNA molecule with a relatively long 5′UTR (untranslated region) and a reading frame of 243 base pairs. The 81 amino acids peptide undergoes significant modifications in the endoplasmic reticulum, including cleavage of membrane- directing region and GPI anchor enhancing peptide tail. The mature peptide is a sequence of 31 amino acids of a molecular weight that ranges from 30 to 75 kDa depending on the attached glycans. It is directed through the GPI anchor to be expressed in lipid-enriched areas that harbor other mucins and signal receptors, known as ‘lipid rafts’ [3].

The human CD24 protein was found on the outer cell membrane of multiple cell lines [4]. It was initially reported to be expressed in the healthy human in B cell populations during specific phases of maturation and absent on mature plasma cells. It was then shown to be expressed on other immune cells including T cells and dendritic cells, during regulatory phases of innate immune response [5]. The commercial availability of an anti-CD24 monoclonal antibody for tissue staining and the development of microarray expression assays lead to an influx of research work finding CD24 overexpression in various human malignancies [6]. This includes a subset of hematologic malignancies–non-Hodgkin B-cell lymphomas, acute lymphoblastic leukemia (ALL) and high-risk acute myeloid leukemia (AML)–but more abundant in carcinoma tumors, including breast, ovarian, colon, hepatocellular, prostate, pancreatic, non-small cell lung, bladder and prostate cancers and others. It has no physiologic expression in the healthy adjacent tissues. In the heart, Chen et al. also reported overexpression of CD24 in cardiac adenocarcinoma tissue, but not in the adjacent mucosa [7]. CD24 was found to be expressed at early stages of the malignant transformation, like intestinal polyps [8], and to confer poorer prognosis [9,10,11]. In vitro and animal models show the significance of CD24 expression in tumor initiation and cell proliferation, drug- resistance, adhesion to vascular endothelium and metastases formation. More recently, CD24 was shown to have an intriguing role in tumor evasion from phagocytosis, hypothesized to act as a ‘don’t eat me signal’ [12].

The functional role in signal pathways and cell communication of a small peptide with no transmembrane domain remains challenging to decipher. Several protein ligands and binding molecules were identified over the years. CD24 can interact with important adhesion molecules, including P-selectin, E-selectin, and L1CAM (CD171) and activate integrins, thus perhaps participating in leukocyte extravasation in inflammation and cancer metastasis. It was more recently shown to bind the immune cell receptors Siglec-5 and Siglec-10 that have an immune- inhibitory role and possibly anti- phagocytic [6]. Intracellular pathways were shown to be activated in response to changes in CD24 expression, directly or indirectly, including CXCR-4, Src or PI3K/Akt kinase proto-oncogenes. Directed biological therapies that block CD24 are thus studied as promising novel cancer treatment modalities, using monoclonal antibodies, chimeric molecules or siRNA [13,14,15,16]. We would suggest that exploring the role of CD24 in cardiovascular disease processes may lead to targeted drug development in our research field as well.

## 2. CD24 as a Marker for Progenitor Cells and Its Implications for Post-Ischemic Myocardial Regeneration

Studied most extensively in the field of cancer research, CD24 is suggested to be a marker for circulating cancer stem cells (CSCs), a small subset of cancer cells that retains differentiation potency and low proliferative profile. CRCs are defined as responsible for tumor initiation, metastases and resistance to most cancer therapies that target cell proliferation. Studying CRCs in breast carcinoma, Al-Hajj et al. suggested that CD44^+^CD24^–^ identified cells with preserved potency and tumor initiating ability while CD24 expression marks epithelial- to- mesenchymal transition (EMT) [17]. Following their lead, other studies in different cancer types found CD24 expression to be a marker for the CSCs themselves, overexpressed on cells that maintain tumor initiating abilities [18,19,20,21]. These findings are of a particular interest to cardiovascular research. The embryonic cellular pathways associated with CSCs are similar to the fingerprint of mesenchymal transition that characterizes cardiac stem cells. CD24^+^ CSCs were shown to have active Notch pathway signaling and high expression of *SOX-2*, *OCT-4* and *NANOG* [6,22], accepted descriptors of successful in vitro derivation of cardiac mesenchymal stem cells (MSCs). *CD24* overexpression was shown to trigger Notch-1 signaling and induce EMT [23].

Human cardiomyocytes respond to injury by hypertrophy and proliferation of local supportive tissues that lead to scarring rather than regeneration of contractile cells that lack a sufficient ability to proliferate [24]. Pilot studies suggest that direct myocardial injection of MSCs from autologous de-differentiated source may assist in post-ischemic regeneration [25]. In children too, using bone-marrow derived MSCs administered intra-coronary to infants with hypoplastic left heart syndrome (HLHS) is trialed with the goal to support recovery and preserve of systolic function in the single right ventricle [26]. Improved and reliable methodologies to identify circulating MSCs or produce them in the lab are studied and new surface markers are in need. In describing a new protocol for deriving MSCs from human embryonic stem cells (hESCs), Lian et al. [27] used gene expression and cell sorting analysis to prove that CD24 is a reliable marker for pluripotent hESCs. They showed that CD24 expression is eliminated in successful MSC differentiation while continues to be positive on other cell lines that are inaccurately derived from hESCs, and thus it is a valuable selection marker. Supporting this conclusion, perhaps, Da Sacco et al. [28] identified CD24^+^ cells in human amniotic fluid to be metanephric progenitor mesenchymal cells.

Valente et al. [29] showed that the adult mouse has a small subset of immature cardiomyocytes that express HAS (mouse CD24 analogue) and have some stem characteristics and EMT potential. These cells are enriched locally post-MI. This is of special interest since unlike human, young mice are known to maintain the ability for myocardial regeneration post-MI. HSA^+^ cells were found in high numbers in the mouse fetal heart, and their percentage among cardiomyocytes declined through pregnancy. Caveolin-3 was used as a marker for maturing contractile cells and its expression increased with the decrease in the number of HSA^+^ cells. HSA^+^ cells at all stages, maintained proliferative capacities, even after birth and when still positive for Caveolin. In contrast, HAS^–^ cells were all out of the proliferative cycle. The HSA^+^ cell subset in the adult mice expressed significantly less genes associated with contractile elements proteins and no genes for proliferation of extracellular matrix. In their expression profile, they shared the highest molecular similarity to embryonic cardiomyocytes. These cells comprised less than 1% of the cardiomyocytes the healthy mouse but expanded up to about 3% after ischemic injury. This suggests that CD24 may be a marker for cells that are capable to lead myocardial regeneration, and possibly derived and enriched in vitro. It should be studied whether CD24 has an active role in regeneration and is increased in response to ischemic injury to promote EMT. In that case, local delivery of CD24 may be therapeutic as a mean to amplify tissue regeneration.

Garcia et al. [30] suggested a different involvement of CD24 in post-MI regeneration in rat model. They suggested that regeneration after ischemic injury is triggered by angiogenesis to the injured area, triggered by vascular endothelial growth factor (VEGF), that then attracts the invasion and expansion of endothelial cells and myocytes. It is proposed that VEGF expression in the adult rat is insufficient for successful regeneration. The authors created a model for acute MI by surgical ligation of the left anterior descending (LAD) coronary artery to test the response to treatment with local injection of *VEGF_165_* cDNA plasmid. By immunohistochemistry, they showed CD24 overexpression in rats treated with VEGF gene delivery compared to the controls. They found this expression a marker for new endothelial cells rather than mesenchymal cells, that were also showed to invade the injured tissue. They found that VEGF overexpression led to decreased apoptosis in the injured tissue and improved ventricular contractility by echocardiogram.

## 3. CD24 in Autoimmune Conditions and Dilated Cardiomyopathy

Since its initial definition as a lymphocytic progenitor marker, studies have shown that CD24 has an active role in propagation and discrimination of the immune response [31]. It is expressed on expanding lymphocyte populations during the maturation of the inflammatory process, possibly inhibiting erroneous activation. CD24 was shown to be a co-stimulator to pathogenic CD4^+^ and CD8^+^ T-cell in various autoimmune conditions, including encephalomyelitis, thyroiditis, giant cell arteritis, multiple sclerosis and others [32]. Polymorphisms or deletions in the *CD24* gene are associated with increased risk for autoimmune conditions, including lupus, giant cell arteritis and multiple sclerosis. In the study of rheumatoid arthritis, patients were found to have lower numbers of CD24^high^CD38^high^ B cells compared to healthy individuals, associated with lesser T cell differentiation to T- regulatory cells and amelioration of auto-inflammation [33]. The specific ligands and mechanisms of CD24 immune modulations are unknown, and several interactions may occur simultaneously as it expresses various B-cells, T-cells and antigen presenting cells (APC). The family of sialic acid-binding immunoglobulin-like lectins (Siglec) are surface receptors that are primarily expressed on hematopoietic cells and considered to be immune suppressors. The study by Barkal et al. [12] was a significant breakthrough in the study of CD24, showing that as it is expressed on breast and ovarian cancer cells, it binds to the immune suppressor receptor Siglec-10 on macrophages to evade recognition by the innate immune system. Chen et al. [34] used a mouse model of liver injury and showed that CD24 binds Siglec-G (the mouse homologue of Siglec-10), and protects from propagation of tissue inflammation through inhibition of NF-κB. They suggested that the CD24-Siglec G binding specifically counters Toll- like receptor dependent response to non-pathogen pro-inflammatory components of damaged cells (danger associated molecular pattern, DAMPs) and thus prevents chronic inflammation. They also suggested that some bacterial strains produce sialidases that recognize and disrupt the CD24-Siglec complex which permits the deterioration to septic response [35]. The chimeric human CD24-Fc protein, a novel immune modulator drug, is suggested to act by potentiating the CD24 -Siglec-10 activating complex and prevent chronic inflammation. Recent clinical studies showed it effective in preventing the progression of HIV infected rhesus monkeys to AIDS [36] and clinical trials in human are now underway for patients with multiple sclerosis or graft-vs-host disease.

Dilated cardiomyopathy (DCM) is an irreversible progressive state of left ventricular dilation and contractile dysfunction with high morbidity and mortality. For non-ischemic DCM, mutations in genes that encode for contractile elements account for up to 35% of presenting cases. Acquired causes may include viral myocarditis, hypertension, cardiotoxic drugs or endocrine conditions. Still, it is estimated that at least a third of the cases are idiopathic [37]. Recent research suggests that chronic myocardial inflammation may be a cause of idiopathic cardiomyopathy. Pro- inflammatory cytokines were shown to have a direct effect on the myocardium: TNF-α triggers apoptosis of cardiomyocytes, and together with IL-1, enhances fibrotic activity by tissue fibroblasts, and IL-6 increases cardiomyocyte stiffness [38]. Myocardial stretch, by itself, triggers Toll receptor and activation of matrix metalloproteases, perpetuating inflammation and fibrosis [38]. Evidence supports a dominant autoimmune nature to tissue inflammation found in DCM as viral genome is not found in most tissue biopsies with presence of immune infiltrates. Furthermore, DCM is common in patients with systemic autoimmune conditions, infiltrative and non-infiltrative; similarly, circulating autoimmune antibodies, systemic or cardiac- specific, are commonly detectable in patients with ‘idiopathic’ DCM and their relatives [39].

Regulatory B-cells (B-Reg) are a subset of circulating differentiated B-cells that are IL-10 secreting. IL-10 is an important anti-inflammatory chemokine in cardiac immunobiology that associated with the inhibition of pro-inflammatory mediators TNF-α and INF-γ that stimulate fibroblasts to produce and deposit collagen leading to permanent myocardial remodeling and scarring [40]. Jiao et al. studied the immune profile of DCM patients and found that the B-Reg subpopulation secreting IL-10 are CD24^high^CD27^+^ cells. The frequency of these circulating CD24^high^CD27^+^ cells was significantly lower in DCM patients than in healthy controls. Additionally, CD24^high^CD27^+^ B cells from DCM patients showed reduced ability to suppress TNF-α secretion from conventional T cells. The frequency of B-Reg cells correlated with left ventricular ejection fraction by echocardiogram and negatively correlated with NT-proBNP (N- terminal brain natriuretic pro-peptide), a marker for elevated end- diastolic pressure and heart failure. Heidecker et al. looked at gender differences in gene expression in patients with DCM. They found that *CD24* expression to be higher in male patients and hypothesized a causative associated to the lower risk of autoimmune condition that involve myocarditis in males compared to females [41].

Regulatory CD24^high^CD27^+^ B cells may also play a dominant role in chronic vasculitis. For instance, CD24^high^CD27^+^ B cells are shown to be reduced in patients with active anti- neutrophil cytoplasmic autoantibody- associated (ANCA) vasculitis compared to those in remission [42]. ANCA vasculitis rarely involves the coronary arteries and the role of T-Reg cells in other vasculitis, such as Takayasu (that often involves the coronary arteries), is controversial. Kawasaki disease, a childhood vasculitis, shows predilection for inflammation of the coronary arteries, occasionally with myocardial involvement. B-cell phenotyping in patients with Kawasaki disease show inconsistent and contradictory findings and, to our knowledge, did not include *CD24* expression analysis [43].

## 4. Study of Systemic Inflammation and Metabolic Syndrome

Metabolic syndrome describes the common constellation of individual risk factor for aberration in adipogenesis and decreased response to insulin that confers high risk for end organ damage, including coronary artery atherosclerotic disease and acute MI, cardiac arrest and heart failure. The suggested pathophysiology is a state of chronic inflammation triggered by hyperinsulinism and hyperglycemia, driven by TNF-α and associated cytokines. Specifically, CD24 has been shown to be a marker of pre- adipocytes while its expression is lost in the mature adipose tissue [44]. Smith et al. [45] investigated its role in adipogenesis and found that *CD24* is specifically expressed on pre-adipocytes that mature to constitute white adipose tissue. The same group [46] showed that CD24 knockout male mice had a significant reduction in adipose tissue mass as a result of decreased cell size (lesser adipocyte hypertrophy). They also showed improved glucose- uptake in response to insulin. In addition, much like in DCM and vasculitis syndromes, Strom et al. [47] showed a protective role to CD24^high^ IL-10 secreting B-regulatory cells in ApoE -/- mice model of atherosclerosis. They found that enrichment of this cell population is associated with neointimal proliferation and progression of atherosclerosis regardless of cholesterol level. They suggest that this population overexpresses CD40L and is expanded in lymph nodes in response to activation of the pro-inflammatory CD40 pathway.

## 5. CD24 as Target for COVID-19 Therapy: An Opportunity for Discovering Novel Cardiac Therapies?

The outbreak of the COVID-19 pandemic raised a special interest in CD24 as an immune modulator. A recent clinical trial (NCT0431704) that was completed in September 2020, proved the chimeric protein CD24-Fc (SACCOVID^TM^, OncoImmune, Rockville, MD, USA) to be an effective therapy in treating critically ill patients with COVID-19 pneumonia when given in conjunction with the anti-viral drug, Remdesivir. This suggests that CD24-Fc may have a specific immune modulatory role that is superior to older generations of anti-inflammatory medications.

In cardiovascular research, a new and specific immune modulator may have clinical significance, not only in treating some complications of acute SARS-CoV-2 infection, but also of the emerging post-viral illness. Particularly in pediatric populations, the post-viral auto-inflammatory condition, MIS-C (multisystem inflammatory syndrome in children, or MIS-A in adults) is a perplexing new entity. Children often present with left ventricular dilation and myocardial dysfunction (66%), pulmonary edema without evidence of pneumonia, and occasionally valvulopathy and coronary artery involvement similar to that of Kawasaki disease [48]. Most patients respond to IVIG (as in Kawasaki) and/or to systemic corticosteroids. Inflammatory markers such CRP, D-Dimer and fibrinogen are significantly elevated at presentation. The pathophysiology is still speculative and thought to involve erroneous auto-immune activation perhaps in a mechanism of molecular mimicry. Direct viral infection to the myocardium is difficult to rule out, yet unlike other forms of viral myocarditis, patients often have laboratory evidence of cytokine activation and auto-antibodies and present with shock and end organ dysfunction that are disproportionate to the degree of cardiac dysfunction alone [49]. Additionally, there is lack of evidence that coronaviruses are cardiotopic and no clear benefit to anti-viral medications [48].

Newell et al. [50] sought to profile the expansion of memory B-cells in COVID-19 patients at the convalescent stage to correlate with positive outcome and define effective humoral immunity. They found large variability in the number and type of memory B-cell populations among individual shortly after SARS-CoV-19 infection. Overall, patients with more robust memory response showed shorter symptom duration, indicative, perhaps, of improved acute phase immunity. They found CD24^+^ ‘class- switched’ memory B cells in all patients, responsible for the production of IgG1, IgG2 and IgG4. They showed that IgG1 antibodies, anti-spike RBD (receptor binding domain, that assists in binding ACE2 receptor in host cells) were at much higher levels in patients with active ‘unswithced’ IgM^+^ memory B-cells. These findings are interesting for the study of MIS-C: it was previously shown that CD24 is nondetectable in the early stage of B-cell maturation until the first D-J rearrangement. It is possible, though still unproven, that CD24 expression marks an early maturation of plasma cells that is less specific in its resulting antibody profile, triggering ‘molecular mimicry’ auto-inflammation.

## 6. Conclusions and Future Directions

CD24 is a membrane protein that raises a significant interest as a surface marker and signal transduction molecule in various biological pathways. CD24 expression has a positive effect in suppressing chronic inflammation and promoting tissue regeneration. As we learn that myocardial ischemia and heart failure are propagated by aberrant local immune response, the role of CD24 in the development of immune tolerance, perhaps through augmentation of regulatory B-cells sub- populations, makes it an interesting new focus to study. As novel therapies that target CD24 show promising results, an opportunity for considerable work is offered to researchers to study and use the protein in cardiovascular research and drug development.
